# Pneumaturia and faecaluria: Symptoms leading to a life-saving diagnosis

**DOI:** 10.1016/j.amsu.2022.103967

**Published:** 2022-06-11

**Authors:** Aye Aye Wynn, Khine San Yin, Nang Khin Mya, Nornazirah Azizan, Andee Dzulkarnaen Zakaria, Firdaus Hayati

**Affiliations:** a Department of Pathology and Microbiology, Faculty of Medicine and Health Sciences, Universiti Malaysia Sabah, Kota Kinabalu, Sabah, Malaysia; b Department of Pathology, University of Medicine 1, Yangon, Myanmar; c Department of Surgery, School of Medical Sciences, Universiti Sains Malaysia, Kelantan, Malaysia; d Department of Surgery, Faculty of Medicine and Health Sciences, Universiti Malaysia Sabah, Kota Kinabalu, Sabah, Malaysia

**Keywords:** Bladder, Colon, Colovesical fistula, Diverticulitis

## Abstract

**Background:**

Colonic diverticulosis is often asymptomatic, but it can complicate bleeding, abscess and stricture. Pneumaturia and faecaluria are unexpected manifestations of colonic diverticulitis complicated by colovesical fistula formation.

**Case presentation:**

This case report highlighted a 57-year-old man who presented with lower abdominal pain which was associated with pneumaturia and fecaluria. He was diagnosed with colovesical fistula after being misdiagnosed with other diseases from various clinic visits. Direct fluoroscopy was performed and a diverticular fistula of the sigmoid colon was confirmed by computed tomography of the abdomen. Hartmann's procedure and resection of the posterior wall of the bladder were resected.

**Conclusion:**

Pneumaturia and faecaluria are common but distinct manifestations of complicated diverticular diseases. Attention should be paid to general practitioners to achieve proper referral, hence early treatment and prevention of disease-related complications.

## Highlights

• Colonic diverticulosis can be complicated by bleeding and stricture including fistula formation.
• Pneumaturia and faecaluria are unexpected manifestations of colovesical fistula.
• General practitioners should be aware of pneumaturia and faecaluria to reduce diverticulitis-related morbidity.

## 1 Introduction

Diverticulosis is a common disease affecting Asians with a prevalence of up to 25% [1]. It is commonly located in the sigmoid colon (75%) [2]. Colonic diverticulosis is often asymptomatic. Among those affected, 4–15% developed inflammation of the diverticulum, hence it is called diverticulitis [1]. However, it can present in different stages of complications such as bleeding, abscess and stricture, including fistula formation to the nearby structures.

The abnormal communication with the nearby structures will manifest a different presentation. When it involves the bladder, various clinical conditions will be manifested. Among those, patients can develop pneumaturia: a passage of gas in the urine forming bubbles; faecaluria: a passage of faeces in the urine; and recurrent urinary tract infection due to ascending infection. Besides diverticular fistula, other causes include diseases affecting the urinary system such as gas-forming urinary tract infection, emphysematous pyelonephritis, renal tumours or urinary catheterization. It is a challenging problem for general practitioners as these alarming presentations can be the presentations of many other disorders. We describe a 57-year-old man who presented with lower abdominal pain, pneumaturia and fecaluria in which a diagnosis of a colovesical fistula was made. This work has been reported in line with the SCARE criteria [3].

## 2 Case presentation

A 57-year-old man presented to the surgical outpatient department with a complaint of air bubbles and faecal matter in the urine. He had been suffering from lower abdominal discomfort for the last two years with occasional dysenteric symptoms. He went to a general practitioner and was treated with antibiotics for the gastrointestinal symptoms which gave him only temporary relief. He continued his work abroad with some available medications but the symptoms had worsened. He took a consultation with another doctor who prescribed steroids for the suspicion of ulcerative colitis. Five months after the start of symptoms, he developed dysuria and then he was treated for urinary tract infection with benign prostate hyperplasia. He still noticed the pneumaturia and fecaluria despite the medications. He was once referred to a psychiatrist for his bizarre symptoms. After 6 months, he was only advised to take an abdominal CT scan and colonoscopy.

**Fig. 1:**
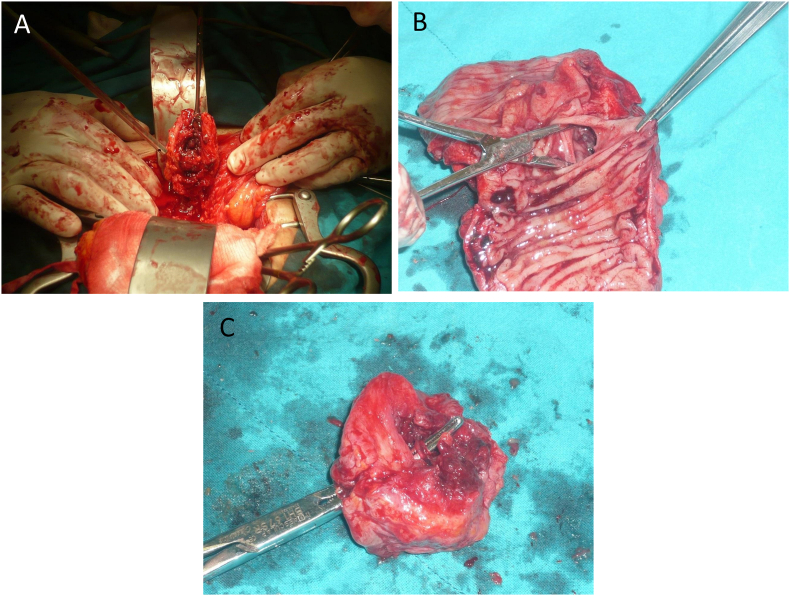
(A) Intraoperative finding of bladder fistula. (B) Presence of fistula on the mucosal surface of the colon. (C) Resected part of connecting fistula in the bladder.

**Fig. 2:**
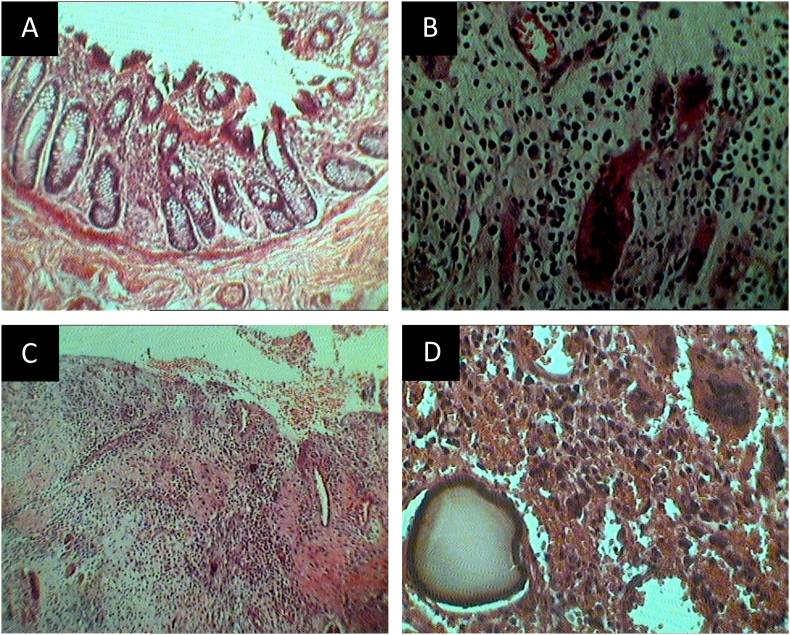
(A) Section from the colonic fistula site shows slightly fibrotic submucosa with surface erosion, (Hematoxylin and eosin, original magnification ×10). (B) Photomicrograph shows the presence of infiltrating chronic inflammatory cells with engorged blood vessels within the colonic wall. (Hematoxylin and eosin, original magnification ×20). (C) Section from the bladder showing eruption of the mucosal surface and dense mixed acute and chronic inflammatory cells infiltrate. (Hematoxylin and eosin, original magnification ×4). (D) Foreign body reaction with the presence of chronic inflammatory cells and foreign body type multinucleated giant cells. (Hematoxylin and eosin, original magnification ×20).

Direct fluoroscopy was performed and a diverticular fistula of the sigmoid colon was diagnosed. Colonoscopy revealed no malignant involvement. The condition was then confirmed by computed tomography (CT) of the abdomen. A laparotomy was performed and a fistula was visualized between the bladder and sigmoid colon (Fig. 1A-C). Hartmann's procedure was performed in which the sigmoid colon and the posterior wall of the bladder were resected and sent to the pathology laboratory for histological examination. Immediate postoperative days were uneventful and the patient was discharged from the hospital on day 10. Histology revealed chronic inflammatory cells infiltration of the intramural colonic wall (Fig. 2A) with the area of submucosal fibrosis (Fig. 2B). The bladder wall is densely infiltrated by mixed acute and chronic inflammatory cells (Fig. 2C) with foreign body reactions of scattered foreign body type giant cells (Fig. 2D). At the 6-month follow up, the patient was symptom-free and doing well.

## 3 Discussion

Any medical practitioner undoubtedly must have gone through the complex cases thoroughly before coming to a final diagnosis especially if the symptoms persist. The possibility of extrapulmonary tuberculosis must be ruled out, especially in this endemic region [4]. Left-sided abdominal pain with faecaluria must exclude colonic malignancy as well [5]. In this case, the patient suffered a stormy journey after being referred from one clinic to another. He was even misdiagnosed with ulcerative colitis, urinary tract infection, benign prostatic hyperplasia and even suspected psychiatric illness requiring a referral to a mental hospital. Ideally, such a presentation must be referred to the tertiary centre for proper assessment.

Classic symptoms of acute diverticulitis are fever, left lower abdominal pain and tenderness, in addition to an increase in markers of acute inflammation [6]. In general, the minority of the cases with diverticulitis develop a colovesical fistula presenting with urinary tract symptoms such as haematuria and pneumaturia. Pneumaturia is the most common symptom occurring in up to 70% of cases [7]. On certain occasions, colovesical fistula also can manifest as faecaluria. Given direct communication with the faecal materials, the likelihood for the patients to suffer from urinary tract infection is deemed high.

On imaging, fistula can be detected either by direct (insertion of contrast through the fistulous tract) or indirect (the communication of the organs or by air detection in the fistulous tract) visualization. In colovesical fistula, only indirect contrast visualization is applicable. CT scan can portray endoluminal air, mural thickening, mucosal hyperemia, tethering of adjacent thick-walled bowel as well as the presence of pericolic fat streakiness [8]. Magnetic resonance imaging is superior to CT scan as the fistula can be visualized at T2–WI with fat suppression sequence and detect a coexisting abscess via DWI with high specificity [8]. Invasive modalities such as cystoscopy and colonoscopy can be helpful to detect fistulous openings.

Colovesical fistulas caused by complicated acute diverticulitis are mainly treated by surgery. At first, medical therapy including bowel rest and empirical antibiotic therapy may be implicated in fistula of non-malignant origin. This indicates that it is extremely crucial to exclude malignancy in such cases. At present, laparoscopic surgery for colovesical fistula offers equal safety and feasibility as compared to open surgery, especially in a high volume laparoscopic colorectal surgery centre [9]. Laparoscopy provides potential advantages namely low incidence of surgical site infections and medical complications.

## 4 Conclusion

Pneumaturia and faecaluria are the common but distinct presences of a variety of diseases ranging from simple urinary tract infections to complicated diverticular diseases. This case report stressed the importance of thorough clinical history and physical examination aided by radiographic investigations to get an early diagnosis of diverticulitis. General practitioners and physicians should be aware of pneumaturia and faecaluria in clinical practice to reduce acute diverticulitis-related morbidity.

## Ethical approval

Ethical approval was obtained from the local institution.

## Sources of funding

None

## Authors’ contributions

AAW - manuscript preparation, KSY - data collection. NKM - literature search, NA - literature search, ADZ - final review, FH - final review

## Registration of research studies

No ethical clearance required as it only involves case report.

## Guarantor

Firdaus Hayati

## Consent

Written informed consent was obtained from the patient for publication of this case report and accompanying images. Permission was also obtained from local administrators. A copy of the written consent is available on request.

## Provenance and peer review

Not commissioned, externally peer-reviewed

## Declaration of competing interest

The authors declare that there are no conflicts of interest.
